# To guide or to follow? Teaching visual problem solving at the workplace

**DOI:** 10.1007/s10459-018-9842-1

**Published:** 2018-07-18

**Authors:** Thomas Jaarsma, Henny P. A. Boshuizen, Halszka Jarodzka, Jeroen J. G. van Merriënboer

**Affiliations:** 1 Research Institute of Child Development and Education, University of Amsterdam, P.O. Box 15780, 1001 NG Amsterdam, The Netherlands; 2 Centre for Learning Sciences and Technologies, Open University of The Netherlands, Heerlen, The Netherlands; 3 School of Health Professions Education, Maastricht University, Maastricht, The Netherlands

**Keywords:** Visual problem solving, Master-apprentice interaction, Clinical pathology, Dialogue analysis, Clinical training

## Abstract

Visual problem solving is essential to highly visual and knowledge-intensive professional domains such as clinical pathology, which trainees learn by participating in relevant tasks at the workplace (apprenticeship). Proper guidance of the visual problem solving of apprentices by the master is necessary. Interaction and adaptation to the expertise level of the learner are identified as key ingredients of this guidance. This study focuses on the effect of increased participation of the learner in the task on the interaction and adaptation of the guidance by masters. Thirteen unique dyads consisting of a clinical pathologist (master) and a resident (apprentice) discussed and diagnosed six microscope images. Their dialogues were analysed on their content. The dyads were divided in two groups according to the experience of the apprentice. For each dyad, master and apprentice both operated the microscope for half of the cases. Interaction was operationalised as the equal contribution of both master and apprentice to the dialogue. Adaptation was operationalised as the extent to which the content of the dialogues was adapted to the apprentice’s level. The main hypothesis stated that the interaction and adaptation increase when apprentices operate the microscope. Most results confirmed this hypothesis: apprentices contributed more content when participating more and the content of these dialogues better reflected expertise differences of apprentices. Based on these results, it is argued that, for learning visual problem solving in a visual and knowledge-intensive domain, it is not only important to externalise master performance, but also that of the apprentice.

## Introduction

Visual problem solving is an essential skill in professional domains such as medicine and aviation. Professionals in these domains solve problems through the interpretation of domain-specific and complex visualizations. Radiologists, for example, make use of CT-scans to diagnose their patients. Several studies have revealed differences in the knowledge structures and problem solving strategies used by experts and non-experts during these tasks (Jaarsma et al. 2015; Jarodzka et al. 2010; Van Meeuwen et al. 2014). Experts, for example, typically spend more time constructing a problem representation, for which they spend more time on low magnification in the first phase of the diagnostic process (Jaarsma et al. 2016). Additionally, experts tend to analyse cases in a comparative manner, in terms of typicality (Jaarsma et al. 2014). These strategies and knowledge structures do thus not only differ fundamentally with expertise, they are also highly covert and become apparent only in interaction with relevant visual stimuli. This raises the question how these complex cognitive and visual skills are learned and taught.

The medical specialty of clinical pathology provides an interesting case for studying the complex cognitive and visual skills that constitute visual problem solving. Clinical pathologists provide diagnoses based on the microscopic examination of tissue samples, a task that requires the mastery of a unique ‘visual language’. This visual language results from the transformation from tissue sample to microscope image: the tissue is conserved, sliced and colored. These processes result in specific visual characteristics (colours, two-dimensional representations of three-dimensional structures) and may also cause artefacts in the image. Moreover, the image ought to be ‘read’ at different levels of magnification, requiring the skill of microscope navigation. To summarize, in order to provide diagnoses, aspiring clinical pathologists have to obtain domain knowledge to understand the domain-specific visualizations of tissue and to identify abnormalities in them through the appropriate manipulation of the presented image.

The knowledge described in the previous section is obtained by trainees in an apprenticeship-like training programme at the workplace. Typically, observation of expert performance (‘modelling’) is a crucial component of the learning process of apprentices (Collins et al. 1989). However, expert performance is not easily observable in the clinical pathology department: apart from training situations or incidental discussions with colleagues, single-headed light microscopes are used for diagnosing in most pathology laboratories, hiding the diagnostic process from the apprentice’s eyes. With the other components of the visual problem solving process being primarily cognitive (i.e., what information experts obtain from the image, and how they use it for their diagnosis) there is not much opportunity for apprentices to construct a conceptual model of expert performance just by ‘being around’. According to Billett (1996), the solution to this problem is twofold. Firstly, the apprentices need to participate in relevant tasks in the workplace, that is, in the visual problem solving process. Secondly, the master needs to guide apprentices directly in this participation, so that expert knowledge and methods involved in these tasks are externalised and thus can be accessed by the learner. This guidance is responsive to the apprentice’s participation (Billett 2000).

Studies on guidance in tutorial dialogues have shown that the active involvement of the tutee is crucial to the learning that takes place (Chi et al. 2001, 2010). Performance gains of tutees in these studies were highest when they were prompted to reflect or to explain matters. Successful guidance in learning dialogues is thus interactive of nature. This often proves to be a challenge for experts (Chi et al. 2001, 2008; Person and Graesser 2003). Besides interaction, common ground is important both for effective conversation (Clark and Brennan 1991) and for the instruction of learners (Shute and Towle 2003). It is thus important to adapt the dialogue to the level of the apprentice. Several studies have shown that estimating the expertise level of a novice, and thus to adapt a dialogue to that level, is particularly difficult for an expert (Hinds 1999; Nathan and Koedinger 2000; Nückles et al. 2005). Providing the expert with specific information of the knowledge level of the novice, proved to support the adaptation (Nückles et al. 2005).

Being crucial to the effectivity of learning dialogues, the question is how interaction and adaptation could be increased, in order to improve learning gains. From the above, it is obvious that interaction and adaptation benefit from input from the learner. Increased participation in the task could lead to increased input and, hence, to increased interaction and adaptation in learning dialogues that surround this task. This study aims to check this hypothesis by increasing the participation of residents (from now on: apprentices) when collaboratively diagnosing tissue with expert pathologists (from now on: masters). The increase in participation consists of apprentices operating the microscope instead of the master (as in daily practice). The following two hypotheses are central to this study: (1) The interaction of learning dialogues in clinical pathology increases when the apprentice operates the microscope, compared to when it is operated by the master; and (2) The adaptation of learning dialogues in clinical pathology increases when the apprentice operates the microscope, compared to when it is operated by the master.

Interaction is thereby understood as the equal contribution to the dialogue by apprentice and master, both qualitatively and quantitatively. This does not mean that the actual input of master and apprentice is equal—there obviously is a knowledge gap—but that both contributors discuss meaningful content, each to their own capabilities. Adaptation is understood as to whether the content of the dialogue matches the expected expertise level of the apprentice, based on expertise studies among clinical pathologists (Jaarsma et al. 2014; Jaarsma et al. 2015). Reflecting the theory on the cognitive development of medical expertise by Boshuizen and Schmidt (1992, 2008), these studies showed that novices, when diagnosing tissue, rely on great detail and simple heuristics, whereas intermediates used biomedical terms in conclusive reasoning chains. It is thus hypothesized that, when apprentices operate the microscope, learning dialogues better reflect their level of expertise.

## Methods

### Participants and design

The participants in this study were 13 clinical pathologists (M_age_ = 49.54 years, age range 33–63, 4 women) and 13 residents (M_age_ = 28.64 years, age range 24–37, 9 women), who formed unique dyads consisting of a master (clinical pathologist) and an apprentice (resident). Two groups of dyads were created based on the experience of the apprentice. Low-expertise (LE) apprentices (7 dyads) were in their first year of training and/or had no experience with the specific organ yet (colon). High-expertise (HE) apprentices (6 dyads) were in their second year of training or higher, and had experience with diagnosing colon tissue. The participants were recruited from three academic hospitals in the Netherlands on a voluntary basis and were rewarded with a small gift voucher after the experiment. The study was approved of by the department’s ethics committee and all participants gave written informed consent.

This quantified qualitative study was designed to measure the effect of microscope operation (as an operationalisation of participation) on the two constructs, interaction and adaptation. These two constructs each had their own independent variable: conversational role (master or apprentice) for interaction, and the apprentice’s expertise level (low expertise, LE, or high expertise, HE) for adaptation. Both constructs, interaction and adaptation, were operationalised using coding categories of features and elements of the conversations as dependent variables (and three additional descriptive variables for interaction). This set-up led to two three-way interactions: microscope operation*conversational role*coding categories/descriptive variables (for the construct of interaction) and microscope operation*apprentice’s expertise level*coding categories (for the construct of interaction). A more detailed description of the variables, predictions, and hypotheses per construct is provided in the “Data reduction and analysis” section. In addition, an overview is provided in Table 2.

### Materials and apparatus

#### Cases

Each dyad discussed and diagnosed six cases, which were digitally scanned microscope images of colon tissue. The images were obtained from the Atrium Medical Centre in Heerlen, the Netherlands. To ensure an actual learning dialogue, difficult cases were selected (based on the judgment of an expert clinical pathologist). Diagnostic accuracy confirmed the complexity, as none of the cases was correctly diagnosed by all dyads (ranging from 0 to 82%). Cases 3 and 5 were particularly difficult as they contained rare abnormalities (squamous metaplasia and amyloid deposits, respectively). The six cases were presented according to a balanced Latin square. The microscopic images were viewed with the Aperio ImageScope digital microscope (version 11.2.0.780). To simulate the normal training situation—in practice a double-headed light microscope is used—two synchronised monitors were used (resulting in two identical displays).

#### Participants’ background

A demographic questionnaire was used to collect background information on the participants, including sex, age, vision and experience with diagnosing colon tissue and with the digital microscope.

#### Dialogues

The dialogues between the master and apprentice were recorded by cameras with an in-built microphone that were mounted on both monitors.

### Procedure

To simulate practice, the apprentices diagnosed the six cases individually (taking 30–45 min), prior to discussing them with the master. The actual experiment began with the participants completing the demographic questionnaire and being instructed on the experiment’s procedure. As they were not all used to a digital microscope, participants were given time to acquaint themselves with its operation. Every case started with reading out aloud the patient background information by the participant that operated the microscope. The same participant then opened the microscopic image and joint examination was started. Viewing time was unrestricted and each case ended with a diagnosis or—in cases where the dyad could not come to a conclusion—a request for further testing. Participants were instructed to behave as they would do in practice. After three cases microscope operation was alternated.

### Data reduction and analysis

The experiment resulted in 73 transcribed dialogues; five recordings (divided over 2 dyads) failed due to time restrictions or technical problems. All acknowledgments (including ‘hmhm’s’) were included as separate turns, as these were of interest to the study. QSR International’s NVivo 10 software was used for analysis, which automatically attributed dialogue turns to master or apprentice, based on the structure of the transcripts.

The coding scheme was designed both in an inductive and in a deductive manner, following methods known as ‘descriptive coding’ and ‘hypothesis coding’, respectively (Saldana 2009). The hypothesis coding was based on our previous expertise difference studies carried out among clinical pathologists (Jaarsma et al. 2014, 2015, 2016) and formed a considerable part of the codes: among others, components of clinical reasoning such as findings, heuristics, and diagnoses were predetermined codes. However, as the data for this study consist of dialogues instead of thinking aloud, some new codes had to be formed by induction from the data (i.e., the descriptive coding part). For this part of the coding, the first author iteratively drafted initial codes and tested these on a subset of transcripts. After a few iterations a coding scheme was designed, consisting of 32 categories. Inter-rater reliability of this scheme was performed by the first and second author: percent agreement was 67.6%, with a Krippendorff’s alpha of .64. The first author then coded all protocols. Afterwards, codes with a similar content were grouped together to facilitate the interpretation of results, reducing the number of codes to 15. For example, basic findings like tissue structures and specific abnormalities were grouped together, while findings that indicated a comparison with mental schemata (identification of absent features, e.g.) formed another group. Table 1 displays definitions of the categories and, per category, a quote from the data as an example (these quotes have been translated from Dutch to English, as participants all were Dutch-speaking). On top of content, questions were coded, too.

**Table 1:** Categories of verbal data, including definitions and examples

Category	Definition	Example(s)
Givens	Patient background and the available material	*So this is a 76*-*year old woman*
Simple findings	Interpretation of findings, expressed in terms of architecture, colours and shapes, impressions, or specific names	*This is necrosis* *Neatly aligned crypts* *This looks like a flower*
Comparative findings	Findings that are expressed as comparisons to mental images of (normal tissue), including artefacts and absent features	*The crypts look typical* *There is thermic damage to the tissue here* *I do not see any inflammation*
Search tactics	Distinctions between relevant and irrelevant areas or specific features mentioned as search targets	*This is our main area of interest* *We need to look for a desmoplastic reaction*
Diagnosis	Hypotheses, rejected hypotheses and statements on the diagnosis	*So this could be Crohn’s disease* *Not malignant, anyway* *Yes, carcinoma*
Difficulty	Comments on the typicality of a case, or on bringing external knowledge or expertise to solve it	*This is not a very neat polyp* *Let the expert take a look at this one* *I would consult a book to see what this tumor should look like*
Further testing	On stainings as a next step in the diagnostic process	*I would do a CD68 to check this*
Workflow	On (the relation to) previous or subsequent stages of the workflow	*They have taken out a lot of tissue* *I would include that as a note in my report* *This whole thing has to be taken out anyway*
Pathology knowledge	Statements on pathology knowledge	*If it were granular cytoplasm, this would have to be hystiocytes* *We see this often in this age group*
Heuristics	Suboptimal viewing strategies used to obtain information from the image, such as comparisons within a case or with a previous case	*There are more crypts here than in the previous case* *This region looks different* *Immediately focus on architecture in these images*
Other’s knowl. and skills	Reflections on knowledge and skills of the other speaker	*You were close, but had the wrong name in mind*
Own knowl. and skills	Reflections on one’s own knowledge and skills	*I really don’t know anything!*
Navigation and process	Navigation moves and references to a certain location, and the diagnostic process	*Could you please move to the left?* *Look there!* *So, what is our next step here?*
Experimental procedure	On the experimental procedure and set-up, or on what the participant would have done in practice	*Are you seeing the same thing as I am?* *I would not have diagnosed this in practice*
Acknowledgments	All acknowledgments and utterances of disagreement	*Hmhm* *No*

The 15 content categories are the measures for the two constructs, interaction and adaptation. Based on previous studies (Jaarsma et al. 2014, 2015, 2016), predictions could be made for some of these measures (see Table 2). However, these studies did not provide a basis for predictions on all 15 content categories. To fully explore the content of the dialogues, all categories are discussed in the “Results” section. The specific predictions are discussed per construct:

**Table 2:** Constructs, hypotheses, measures, and predictions

Constructs	Interaction		Adaptation	
Definition	Master and apprentice have an equal share in the content of learning dialogues.		The adaptation of the content of the learning dialogues to the expertise level of the apprentice.	
Hypothesis	The share of the apprentice in the contribution of content to the dialogue increases when apprentices operate the microscope, as compared to when it is operated by masters.		Learning dialogues are better adapted to the level of the apprentice when apprentices operate the microscope than when masters operate it.	
Predictions				
*Content categories*	*Operated by master*	*Operated by apprentice*	*Operated by master*	*Operated by apprentice*
Givens	A < M	A = M	LE = HE	LE < HE
Simple findings	A < M	A = M	LE = HE	LE > HE
Comparative findings	A < M	A = M	LE = HE	LE < HE
Search tactics	–	–	–	–
Diagnosis	–	–	LE = HE	LE < HE
Difficulty	–	–	LE = HE	LE < HE
Further testing	–	–	LE = HE	LE < HE
Workflow	–	–	LE = HE	LE < HE
Pathology knowledge	–	–	–	–
Heuristics	A < M	A = M	LE = HE	LE > HE
Other’s knowledge and skills	–	–	–	–
Own knowledge and skills	–	–	LE = HE	LE > HE
Navigation and process	–	–	–	–
Experimental procedure	–	–	–	–
Acknowledgments	A > M	A = M	–	–
*Quantitative measures*				
Coverage by apprentice	*Operated by master ***<** *Operated by apprentice*		–	
Short turns by apprentice	*Operated by master ***>** *Operated by apprentice*		–	
Questions by apprentice	*Operated by master * **< ** *Operated by apprentice*		–	

#### Interaction

To measure the effect of microscope operation on interaction, frequencies of content categories are compared between the master and apprentice. As apprentices do not master the full diagnostic process yet, predictions for interaction only concerned those categories that correspond with their expertise levels: givens, simple and comparative findings, heuristics, and acknowledgments (see Table 2). First, a three-way loglinear analysis (microscope operation*contributor*content categories) was performed to analyse whether microscope interaction affected the content and number of contributions of master and apprentice. Afterwards, separate Chi square analyses on the content categories and contributor (master/apprentice) were performed for both conditions of microscope operation.

In addition, three quantitative measures of interaction were derived from the verbal data: *coverage of the apprentice* (percentage of total words), *questions asked by apprentice*, and *number of short turns by apprentice* (1–3 words). The independent variable in this analysis was microscope operation. Because of the small sample sizes, non-parametric tests were used (Mann–Whitney).

#### Adaptation

To measure the effect of microscope operation on the adaptation of the dialogue to the level of the apprentice, the content of dyads with low-experienced apprentices (LE dyads) was compared with that of dyads with high-experienced apprentices (HE dyads). Based on literature (Jaarsma et al. 2014, 2015, 2016), predictions could be made for a total of nine categories for adaptation (see Table 2). First, a three-way loglinear analysis (microscope operation*apprentice expertise*content categories) was performed to analyse whether microscope interaction affected the content and of LE and HE dyads. Afterwards, separate Chi square analyses on the content categories and apprentice experience (LE/HE) were performed for those cases when masters operated the microscope, and when apprentices operated the microscope.

## Results

The learning dialogues had a median time-on-task of 223 s and a median of 575 words. Microscope operation affected neither the time-on-task (*U *= 597.50, *z *= − .76, *p* = .45), nor the number of words (*U *= 578.00, *z *= − .97, *p* = .33).

This section discusses the results per construct.

### Interaction

#### Content categories

The three-way loglinear analysis produced a model with a likelihood ratio of χ^2^(0) = 0, *p* = 1. This implies that its highest-order interaction (content categories*operator*contributor) was significant, χ^2^(14) = 98.68, *p* < .01. Masters and apprentices thus contributed different content to the dialogue, depending on the microscope operation. To break down this effect, separate Chi squares were conducted for microscope operation by masters and apprentices. The left pane of Table 3 shows the frequencies and significance per contributor per content category.

**Table 3:** Frequencies (n) and standardized residuals (z) of the content categories, for interaction (left) and adaptation (right)

	Interaction								Adaptation							
	Operated by master				Operated by apprentice				Operated by master				Operated by apprentice			
	Apprentice		Master		Apprentice		Master		LE		HE		LE		HE	
	*n*	*z*	*n*	*z*	*n*	*z*	*n*	*z*	*n*	*z*	*n*	*z*	*n*	*z*	*n*	*z*
Givens	44	− 1.17	69	1.09	110	4.38*	36	− 4.36*	51	− 2.52*	62	3.38*	80	− 0.78	66	0.95
Simple findings	251	1.18	251	− 1.1	361	3.77*	234	− 3.75*	353	1.72	149	− 2.31*	344	− 0.65	252	0.79
Comparative findings	65	1.25	55	− 1.16	75	1.55	51	− 1.54	78	0.12	42	− 0.15	85	1.12	41	− 1.36
Search tactics	27	− 1.79	55	1.67	33	− 0.71	42	0.71	50	− 0.36	32	0.48	46	0.17	29	− 0.21
Diagnosis	67	− 2.47*	128	2.30*	94	− 1.26	121	1.25	120	− 0.46	75	0.61	107	− 1.9	108	2.31*
Difficulty	16	− 2.52*	48	2.34*	23	− 0.57	29	0.56	36	− 0.79	28	1.06	20	− 1.99	32	2.42*
Further testing	33	0.01	38	− 0.01	31	− 3.81*	91	3.80*	42	− 0.53	29	0.70	66	− 0.81	56	0.99
Workflow	19	− 1.6	40	1.49	22	− 0.67	29	0.67	19	− 3.06*	40	4.1*	19	− 2.08*	32	2.54*
Pathology knowledge	41	− 5.59*	165	5.20*	59	− 5.04*	167	5.02*	129	− 0.28	77	0.37	143	0.68	83	− 0.83
Heuristics	7	− 2.04*	25	1.90	22	− 0.48	27	0.48	19	− 0.34	13	0.45	44	2.72*	5	− 3.31*
Other’s knowledge and skills	1	–	14	2.10*	0	–	12	2.43*	12	0.77	3	–	8	0.31	4	− 0.38
Own knowledge and skills	50	1.85	33	− 1.72	51	2.05*	26	− 2.04*	59	0.79	24	− 1.05	41	− 0.74	36	0.9
Navigation and process	91	− 1.48	138	1.38	147	− 2.71*	222	2.69*	150	0.25	79	− 0.34	237	1.07	133	− 1.3
Experimental procedure	24	− 0.12	29	0.11	29	1.64	14	− 1.63	44	1.71	9	− 2.29*	19	− 1.32	24	1.61
Acknowledgments	392	6.60*	214	− 6.14*	389	0.89	358	− 0.89	397	0.42	209	− 0.56	478	1.52	268	− 1.85

An asterisk indicates a significant difference (*p* < .05) between actual and expected frequency (i.e., − 1.96 ≥ *z* ≥ 1.96). A negative *z*-value indicates that the frequency is lower than expected, a positive value means it is higher than expected. For frequencies smaller than five, no *z*-value could be calculated

When masters operated the microscope, there was a significant association between the content categories and the contributor, χ^2^(14) = 209.36, *p* < .01. This means that masters and apprentices contributed different content to these dialogues. More in particular, when masters operated the microscope, they contributed more comments on the diagnosis, difficulty of the case, pathology knowledge and the knowledge of the apprentice. Apprentices contributed many turns that fell in the category of ‘acknowledgments’.

**Fig. 1:**
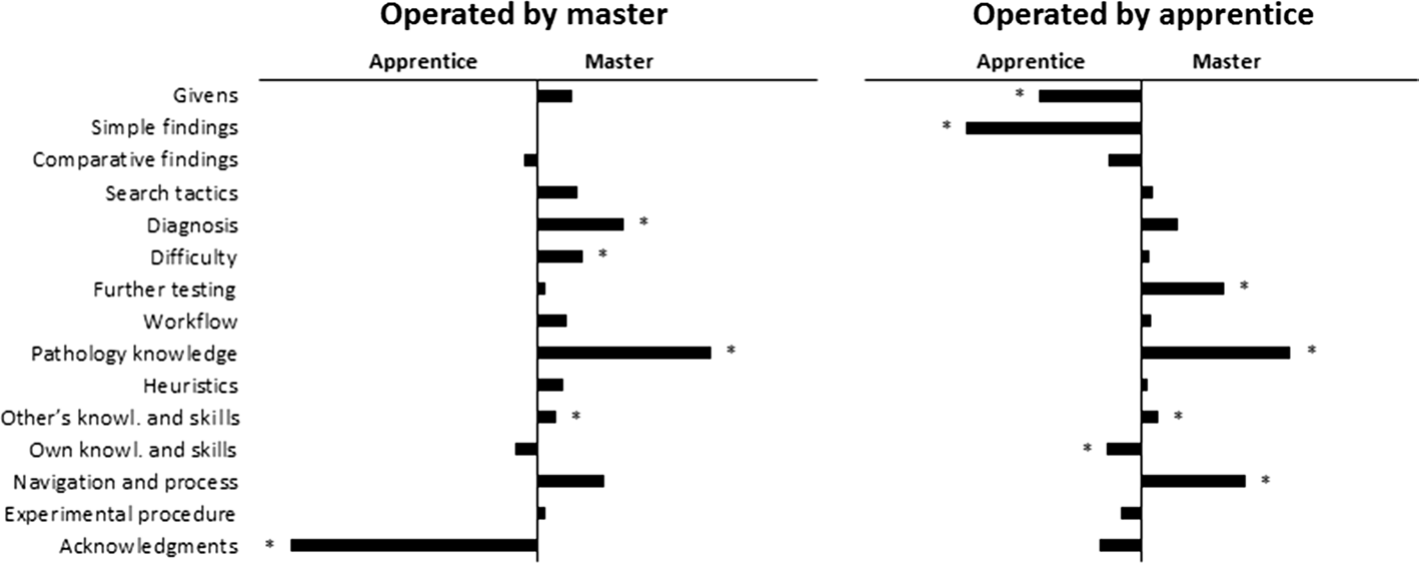
Visualization of the level of interaction between apprentice and master, as a function of microscope operation by masters (left panel) and apprentice (right panel). The bars indicate the difference between the master and apprentice: the smaller, the more equal their contributions were. An asterisk indicates that the frequencies for apprentice and master significantly deviated from their expected frequencies (see Table 3)

When apprentices operated the microscope, there also was a significant association between content categories and the contributor, χ^2^(14) = 198.76, *p* < .01. When operating the microscope, apprentices contributed most comments on the givens of the case, simple and comparative findings, and their own knowledge and skills. Masters contributed most comments on further testing, pathology knowledge, and the experimental procedure. A visualisation of these results is given in Fig. 1.

#### Quantitative measures

Microscope operation affected the coverage of the apprentice (*U* = 414.00, *z* = − 2.78, *p* < .01, *r* = − .33) and the number of questions asked by the apprentice (*U* = 470.00, *z* = − 2.65, *p* = .01, *r* = − .31). When they operated the microscope, apprentices had a larger share in the total words (Mdn = 47.52 vs. Mdn = 41.13) and asked more questions (Mdn = 2.50 vs. Mdn = 1.00) compared to when the master operated the microscope. Microscope operation did not affect the number of short turns by the apprentice (*U* = 547.00, *z* = − 1.82, *p* = .07).

### Adaptation

#### Content categories

The three-way log linear analysis resulted in a model with a likelihood ratio of χ^2^(0) = 0, *p* = 1. This means that the three-way interaction (content categories*operator* apprentice experience) was significant (χ^2^ (14) = 51.94, *p* < .01), indicating that the adaptation of masters to the apprentice was affected by who operated the microscope. To break down this effect, separate Chi square analyses were performed for those sessions operated by the master and by the apprentice. The right pane of Table 3 displays the results of these analyses.

When masters operated the microscope, there was a significant association between the content categories and the apprentice experience, χ^2^(14) = 70.92, *p* < .01. This means that there was different content being discussed by LE and HE dyads. More specifically, HE dyads discussed the givens of the case and the workflow more than LE dyads. The latter discussed more simple findings.

**Fig. 2:**
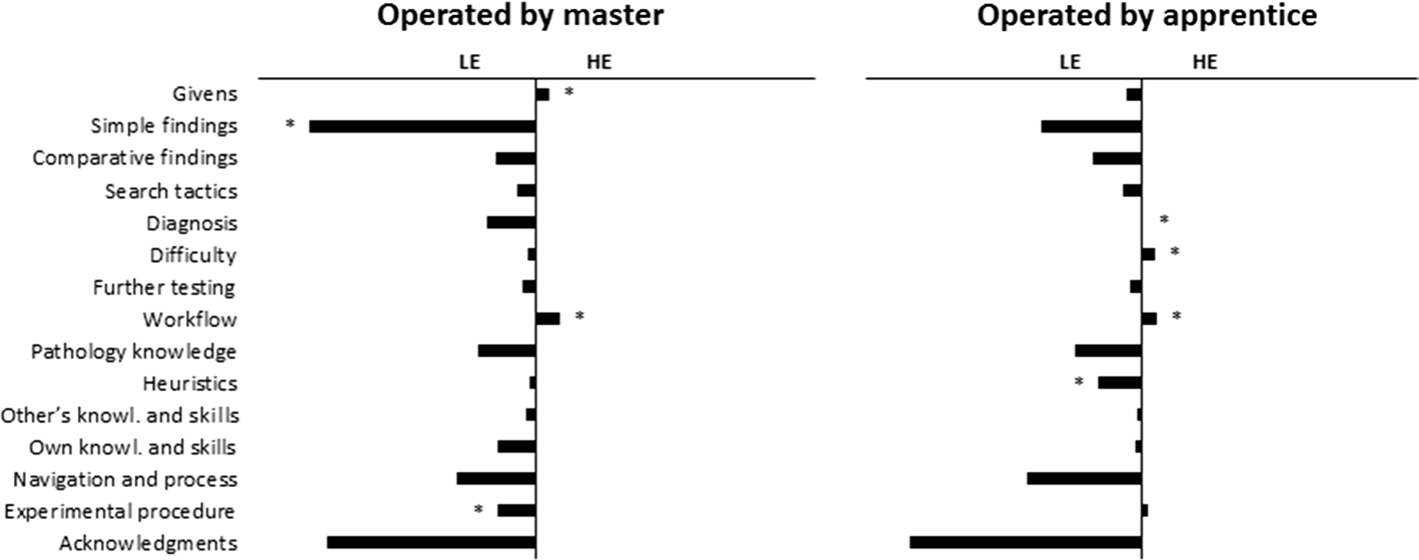
Visualization of the level of adaptation of the content to the level of the apprentice, as a function of microscope operation by masters (left panel) and apprentice (right panel). The bars indicate the difference between LE dyads and HE dyads: the smaller, the more equal the content was in these dyads. An asterisk indicates that the frequencies for LE and HE dyads significantly deviated from their expected frequencies (see Table 3)

When apprentices operated the microscope there also was a significant association between the content categories and the apprentice experience, χ^2^(14) = 86.80, *p* < .01. This implies that different content was discussed by LE and HE dyads in these dialogues: HE dyads discussed diagnoses, case difficulty, further testing, and the workflow more than LE dyads. LE dyads discussed more heuristics. Figure 2 provides a visualisation of these results.

Table 4 shows a fragment of a dialogue between an experienced apprentice and a master, in a case in which the apprentice operated the microscope. It shows the way in which dialogues were segmented and coded. In this fragment, the apprentice is clearly leading the diagnostic process. The master follows the apprentice in his reasoning and asks questions to check the robustness of the apprentice’s statements. The apprentice thus takes a very active role, making the dialogue truly interactive.

**Table 4:** Part of a dialogue between an experienced apprentice and a master, with the apprentice operating the microscope

Sgmnt	Apprentice	Master	Code
1	I think that, because of that localisation in the colon ascendens it is, it corresponds well with a Sessile Serrated Adenoma and it also has the morphological characteristics of that, I believe		*Pathology knowledge*
2	With those horizontally oriented tubes against the basement membrane		*Simple findings*
3	And some occasional branching. With low branches		*Simple findings*
4		Yes, and what are your main criteria to distinguish between a hyperplastic polyp and a Sessile Serrated Adenoma?	*Pathology knowledge*
5	Yes, well, these—to me that is primarily those low-branching tubes		*Pathology knowledge*
6	These, here, horizontal and also like anchors-		*Simple findings*
7		Yes	*Acknowledgment*
8	-or as try squares-		*Simple findings*
9		Yes, but you said ‘branching’. But what you actually mean is-	*Other’s knowledge and skills*
10	Yes, horizontal-		*Acknowledgment*
11		-angulated, hooked	*Acknowledgment*
12	Yes, parts of it are hooked,		*Acknowledgment*
13	but sometimes there is a kind of branching		*Simple findings*
14	And, according to me, a hyperplastic polyp does not branch so low		*Pathology knowledge*
15		And why would it not be dysplastic?	*Diagnosis*
16	I think it matures well towards the surface		*Simple findings*
17	There is some stacking down below,		*Simple findings*
18	but at the surface it is in fact beatifully single-layered		*Simple findings*
19	and also, yes, neither do I find the morphology of the nucleus dysplastic		*Simple findings*
20	There is little anisokaryosis in it		*Simple findings*
21	Little stacking		*Simple findings*

The dialogue is split by segment, with the corresponding codes being given per segment. Note that a dialogue turn can consist of multiple segments

## Discussion

This study tested two hypotheses on the effect of increased apprentice participation in a relevant workplace task on the interaction and adaptation in learning dialogues. Hypothesis 1 stated that the interaction between master and apprentice increases with increased apprentice participation (i.e., apprentices operate the microscope instead of masters). Apprentices were predicted to have a greater share in the dialogue, both in quantity and quality. Most of these predictions were confirmed. When operating the microscope, apprentices contributed more to the dialogue, asked more questions, and were less passive (expressed by number of acknowledgments) than when masters operated the microscope. These results suggest that there was more interaction between master and apprentice in the dialogues with increased apprentice participation.

Hypothesis 2 stated that increased apprentice participation stimulates adaptation of the dialogue to the apprentice’s level, expressed in more basic findings for low-experienced apprentices and more ‘advanced’ content (e.g., diagnoses and their consequences) for high-experienced apprentices. These predictions were mostly confirmed. When apprentices operated the microscope, dialogues with high-experienced apprentices contained more diagnoses, difficulty, further testing, and workflow, while low-experienced apprentices discussed more heuristics. These differences in content correspond with expertise differences between novices and intermediates in clinical pathology (Jaarsma et al. 2014, 2015) and thus suggest adaptation to the level of the apprentice.

Quite remarkably, masters frequently commented on ‘further testing’ when apprentices operated the microscope: more than apprentices did (125 versus 45) and also much more than when they operated the microscope themselves. ‘Further testing’ entails additional tests to confirm or exclude the presence of abnormalities. These tests are thus only requested when no certain diagnosis could be reached based on the available material. This relatively high frequency could mean that masters do not feel certain about their conclusion when they are not acting hands-on themselves. They tried to overcome this by steering the apprentice to relevant areas, based on the high frequency of ‘process’ comments. However, it could be that they did not succeed in verbalising everything they wanted to check, and thus resorted to additional tests.

What do these results teach us for the training of apprentices in clinical pathology and other visual and knowledge-intensive professions? Most importantly, the covertness of visual problem solving expertise does not only count for experts—as was argued in the Introduction—but also for apprentices: Just like it is difficult for apprentices to know what masters actually do, it is difficult for masters to detect what apprentices do. When apprentices’ participation increased, allowing apprecntices to show their ability level, the content of the dialogues changed for the better (i.e., more interaction, more adaptation). The conversation focused more on their expertise, instead of that of the master. An interesting example is the increase in the heuristics that were discussed. Heuristics are rules of thumb on how to make sense of an image, for example to compare certain parts of the image with others. It is very likely that masters are prompted to share such rules of thumb when they actually see how an apprentice goes about diagnosing an image. When the master is controlling the image, this would either require a constant verbalisation by the master of the actions carried out (and why!) or questions from the apprentice to go beyond learning by mere observation. Similarly, the master could also choose to ‘interrupt’ an apprentice’s diagnostic reasoning when the followed routine would not lead to the correct diagnosis. As Moulton et al. (2007) argue, the ability to judge when to let go automatic processing and to adopt more effortful reasoning is a crucial aspect of expertise. This judgment could be trained more effectively when apprentices participate more, offering the master the opportunity to witnesses the diagnostic process of the apprentice in real time and in more detail.

It could thus be valuable for training purposes to externalise the knowledge of apprentices by increasing their participation in meaningful tasks. However, the training of clinical pathologists—as any form of workplace learning—is nested within an operational medical department. Clinical pathologists are diagnosticians just as much as they are educators. To determine whether microscope operation slowed the working process down, a quick analysis of the effect of microscope operation on time-on-task was carried out. When all cases were analysed together, no effect of microscope operation on time-on-task was found. However, when dyads were inspected separately, it turned out that for most dyads (8 out of 13) the cases in which the apprentice drove the microscope had a higher average time-on-task. In addition with more requests for further testing being made when apprentices operate the microscope, it is probably more beneficial for the productivity of the pathology department to have apprentices observe masters, rather than vice versa. However, investing in extra time to increase the participation of pathology residents could lead to more effective training of the future pathologists. As pathology might not be the most time-pressed and—in terms of patient well-being—most precarious medical specialty (as compared to, for example, surgery) it might form an appropriate context to increase learners’ participation. However, when considering the implementation of such a teaching innovation, it is important not to look only at its costs, but also at its benefits. If total training time could be diminished with a slightly decreased workflow, this still might be an efficient intervention.

To determine whether the expected gain in effectivity outweighs the investment of an impaired work process, future studies should focus on the performance gain of increased participation in working tasks. Not measuring performance gain is the most important limitation of this study. Another missing aspect and therefore interesting direction for future studies would be to include the strategies that experts use in their guidance of apprentices. This could reveal which strategies are most effective, and how these differ with the expertise level of apprentices.

To conclude, this study has explored new ground by studying in detail the learning dialogues as they take place at the workplace, in a highly visual and knowledge-intensive domain. Previous studies focused on parts of this process, such as visual but content-free puzzle tasks (Gergle 2006; Richardson et al. 2007), or studies on tutor groups of medical students involved in problem-based learning (Frederiksen 1999; Glenn et al. 1999; Koschmann 1999). However, much more unexplored ground lies ahead of this study. The study has shown that, when teaching highly cognitive and thus covert knowledge in the workplace setting, it is important to externalise not only the master’s expertise, but also that of the apprentice. This insight can be used to improve workplace learning of visual problem solving.
